# Northward expanding resident species benefit from warming winters through increased foraging rates and predator vigilance

**DOI:** 10.1007/s00442-018-4271-7

**Published:** 2018-10-24

**Authors:** Veli-Matti Pakanen, Eveliina Ahonen, Esa Hohtola, Seppo Rytkönen

**Affiliations:** Ecology and Genetics Research Unit, University of Oulu, PO Box 3000, 90014 Oulu, Finland

**Keywords:** Climate change, Non-breeding distribution, Survival, Warming behaviour, Wintering adaptation

## Abstract

Species distributions shift northwards due to climate change, but the ecological mechanisms allowing range expansions are not fully understood. Most studies have concentrated on breeding seasons, but winter warming may also be important. Wintering distributions are restricted by food availability and temperature, which may also interact. Foraging in cold conditions requires adaptations as individuals have to be efficient in foraging, while staying warm and vigilant for predators. When the ambient temperature declines, foraging rates should be reduced due to increased time spent on warming behaviours. In addition, predator vigilance should decline, because more time has to be invested in foraging. Cold weather should limit northward expanding southern species in particular, while northern species should perform better in cold conditions. We tested this by studying temperature responses (between 0 and − 35 °C) among wintering birds at feeders. We compared foraging behaviours of two northward expanding southern species, the great tit (*Parus major*) and the blue tit (*Cyanistes caeruleus*) to a northern species, the willow tit (*Poecile montanus*). Foraging rate and vigilance decreased, and warming behaviour increased when temperatures declined. Importantly, the performance in these traits was poorer in the southern species compared to the willow tit. Furthermore, the response to decreasing temperatures in foraging rates and warming behaviour was stronger in the great tits than willow tits. As the winters become warmer, these mechanisms should increase wintering success of southern species wintering at high latitudes, and lead to higher survival, increased population growth, and consequent range expansion.

**Electronic supplementary material:**

The online version of this article (10.1007/s00442-018-4271-7) contains supplementary material, which is available to authorized users.

## Introduction

Climate change shifts species distributions polewards in most taxa (Thomas and Lennon 1999; Parmesan and Yohe 2003; Brommer et al. 2012; Lehikoinen and Virkkala 2016). To date, most studies have concentrated on breeding season phenological consequences of climate change (e.g., Visser et al. 1998; Vatka et al. 2011, 2014), while wintering ecology is less examined despite shortening winters (Sparks and Menzel 2002) that warm up the most (Jylhä et al. 2009). Warming of winters may be an important factor behind range expansion, because surviving the winter should become easier further north (Maclean et al. 2008; Zuckerberg et al. 2011; Lehikoinen et al. 2013; Fraixedas et al. 2015), but the ecological mechanisms allowing range expansions are not fully understood (Gaston 2009).

Winter is a demanding time in the northern latitudes. At the same time, when food availability is reduced, low temperatures require higher food consumption due to increased energy demands for heat production. For diurnal birds, shortened day lengths limit birds, because they have less time to acquire enough energy for their daily activities and for attaining sufficient fat deposits that ensure survival through the long and cold nights (e.g., Calder and King 1974; Biebach 1996; Koivula et al. 2002; Krams 2002; Krams et al. 2010). Successful wintering in cold conditions where temperatures can decline below − 30 °C requires morphological, physiological, and behavioural adaptations (e.g., Brooks 1968; Biebach 1996), and alleviation in temperature should thus help southern species.

Wintering distributions are restricted to areas where the increased energy requirements of cold weather and metabolic rates do not grow too high (Root 1988, Meehan et al. 2004). Increasing winter temperatures reduce these requirements. Birds maintain their body temperatures partly by a high basal metabolic rate (BMR), which increases during the winter in many species (McKechnie 2008). When temperatures decline below the thermoneutral zone, basal metabolism, activity, and the heat increment of feeding do not produce enough heat (Calder and King 1974), and body temperatures are maintained by shivering thermogenesis (Hohtola et al. 1980; Hohtola 2004). Wintering birds use about 20–40% of their daily energy expenditure on heat production (Weathers et al. 1984; Weathers and Sullivan 1989). Consequently, wintering small birds are forced to forage large parts of the day (Austin 1974; Cooper 2000). Therefore, the ability to forage efficiently should be vital for acquiring energy reserves that ensure survival.

Winter distributions can be further limited by food availability in cases where physiological limitations do not restrict wintering (Repasky 1991; Canterbury 2002). A natural experiment occurs when winter feeding increases food availability and lures species into wintering in more northern locations than enabled by their adaptations. However, temperature seems to be more important than resources in determining wintering distributions (Zuckerberg et al. 2011), and these species of southern origin may still face problems in foraging in the cold environment.

Among birds, general adaptations to reduce energy and heat loss during the winter include finding less windy, warmer, and most profitable feeding and roosting sites (Sulkava 1968; Alatalo 1982; Wachob 1996; Veľký et al. 2010), reducing movements (e.g., Pakanen et al. 2018), using over-night hypothermia (Haftorn 1972; Reinertsen 1983; McKechnie and Lovegrove 2002), ptiloerection, and peripheral vasoconstriction, especially to keep their feet colder than their bodies (Johansen and Bech 1983). Unfeathered parts are an important avenue of heat loss in birds (Steen and Steen 1965, Hill et al. 1980, Yorzinski et al. 2018). Heat loss from legs can be reduced by vasoconstriction, but, at temperatures below freezing, birds have to use postural adjustments to cover the legs with insulating feathers for additional cold defense. This can curtail heat loss by 20–50% (Dawson and Whittow 2000). Using ventral plumage for insulation by raising a foot or crouching may also be necessary to avoid cold bites at very low temperatures. However, such heat loss preventing behaviour may reduce foraging efficiency (Grubb 1978). This behaviour should increase with decreasing temperature, and be more common in the less adapted southern species.

During daily activities, individuals also need to scan the environment to escape depredation (Lendrem 1983; Treves 2000). However, there is a trade-off between time spent vigilant and foraging, which becomes a problem in cold conditions as birds need to increase foraging to meet their daily and over-night energy requirements (Caraco 1974; Hogstad 1988a, 2015 Pravosudov and Grubb 1995; Brodin et al. 2017). Reduced vigilance, in turn, may result in increased predation (Lind and Cresswell 2005). Therefore, wintering birds need to divide their daily activities between foraging, warming, and vigilance, and species that are best adapted to northern conditions should be better able to maintain foraging rates and vigilance in cold conditions, and allocate less time to warming behaviour.

Two temperate species, the great tit (*Parus major*) and the bue tit (*Cyanistes caeruleus*), have extended their breeding and wintering distributions to Northern Fennoscandia in the 20th century (Haartman et al. 1967; Väisänen et al. 1998; Valkama et al. 2011). Great tits reached northern parts of Fennoscandia in the late 1950s (Haftorn 1957; Veistola et al. 1995). Blue tits were very rare in the latter part of the 19th century being present only in the southernmost parts of Finland but extended their range across Finland during the 20th century (Valkama et al. 2011), reaching Oulu in greater numbers in the 1990s. While both species breed in northern Scandinavia, their densities are lower than in the south (Valkama et al. 2011). Winter feeding has influenced the winter distributions of these facultative migrants (Orell 1989; Nowakowski and Vähätalo 2003; Nilsson et al. 2006; Valkama et al. 2011). They commonly coexist with, and visit the same feeders as the willow tit (*Poecile montanus*) that are fully adapted to the northern conditions with territorial flocks that store food for the winter (Ekman 1989). While all species benefit from supplemental food (Lahti et al. 1998; Koivula et al. 1996; Jansson et al. 1981), the temperate species may be poorer in maintaining foraging rates due to lack of adaptations to the cold temperatures.

Here, we compared the performances of great tits and blue tits to the willow tit in the conditions of ample food (at feeders) in Northern Finland under temperatures ranging from 0 to − 35 °C. We used video recordings taken at feeders during winter to examine (1) how temperature affects the proportion of time which they can allocate to processing food (i.e. foraging rates), warming, and heat loss preventing behaviour and predator vigilance? We expected these behaviours to be dependent on temperature in all species, but we expected willow tits to have higher foraging rates and predator vigilance but show less warming behaviour. We further asked (2) whether the above species show different responses to temperature in these traits? As a boreal species, willow tits should be better adapted to cold conditions than the temperate species. Hence, they should be more efficient foragers, they should need to spend less time warming, and they should be able to be more vigilant in cold weather compared to the southern newcomers.

## Materials and methods

### Study populations

Foraging blue tits, great tits, and willow tits were video recorded in northern Finland sites along two temperature isotherms: (1) in Oulu, Hietasaari (65°01′N, 25°28′E), Oulu, Sanginjoki (64°95′N, 26°01′E), and Sonkajärvi (63°40′N, 27°31′E), and (2) at Ranua (65°92′N, 26°56′E) further in the north. Data from Oulu and Sonkajärvi were collected in 2010–2012 and data from Ranua in 2011–2012. Feeding sites were similar in terms of safe areas for reaching the feeders and for foraging. Thus, we consider the feeding sites to be equal in terms of predation risk effects on foraging and vigilance behaviour (Hogstad 1988b; Ekman 1987).

### Data collection and analysis

We used Canon PowerShot A580 and Panasonic Lumix DMC-FZ18 digital cameras in recording videos of foraging tits, which were supplied with sunflower seeds and peanuts. We prevented disturbance to birds by filming from a car, through house windows or behind natural shelters from a distance of 5–10 m. We filmed foraging during daytime (9:00–15:00) when light conditions allowed filming. Length of the videos included in the study ranged from 10 s to a few minutes. Temperatures varied between 0 °C and − 35 °C. The data consisted of 241 video recordings (Oulu 115, Sonkajärvi 7, Ranua 119). There were videos from 34 blue tits, 152 great tits, and 55 willow tits that were similar in length and in ambient temperatures (Online resource 1). However, the coldest − 35 °C temperatures were recorded only for great tits. We, therefore, reran the statistical models (see below) with data spanning until − 30 °C to see if the coefficients stayed similar.

Videos were analysed using the program Anvil 5 (Kipp 2001), which can be used to determine time-budgets and quantify time-coded behavioural events. Starting from the time a bird fetched a seed/nut, we registered and marked the following parameters. (1) *Foraging rate* was measured as the proportion of time used in processing seeds from the overall time spent at the feeding site. We registered the starting and ending times of foraging, and the starting and ending times of pauses between foraging. Tits keep the seed in their toes when they process it with their bill (Yince 1964). (2) *Warming behaviour* was measured as the proportion of time that the seed was held in the beak. (3) *Heat loss preventing behaviour* was measured by whether the birds kept their feet most of the observed time under their feathers (coded 1) or not (coded 0). We used a dichotomous (0/1) variable, because the distribution was extremely discrete. In cold conditions, tits have to warm their toes inside their plumage (Dawson and Whittow 2000), and this was observed to affect foraging, either by totally disrupting it (indicated by holding the seed in the bill), or making it more difficult. (4) *Vigilance* was registered in timeline as events when the bird presumably scanned for possible predators by raising its head and stopped foraging activities.

We used linear mixed models (LMM) in program R (R Development Core Team 2017) to analyse foraging rates, warming behaviour (the proportion of time seed was kept in the bill) and vigilance, and generalized linear mixed models (GLMM; binomial errors, logit link) to analyse heat loss preventing behaviour (legs hidden among feathers most of the time or not). We included species (willow tit as reference) and temperature during observation as fixed effects. The sites were divided into two temperature isotherms and site was included as a random effect with random annual slopes. We centred continuous variables to enable the proper interpretation of interaction models (Schielzeth 2010). We constructed several models starting with an intercept-model, and added temperature, species, and their interactions, consecutively, to find the best model explaining the variation in the studied behaviours. Model comparison was based on the Akaike Information Criterion (Burnham and Anderson 2002). In all cases, we were able to find the best model that was over two AIC-units better than the next best model. We did not individually mark birds, which meant that we could not include individual as a random factor. However, on the basis of a few colour ringed individuals, we could see that the within-individual variation was clearly larger than the between individual variation in the observed temperature scale. This means that the effect of temperature on behaviour should not be biased, even though some observations were made from the same individuals (Leger and Didrichsons 1994). Lack of data prevented us from including dominance rank, sex, or age of individuals in our models, even though they may be linked to vigilance behaviour (Krams 1998).

## Results

### Foraging rates

**Fig. 1:**
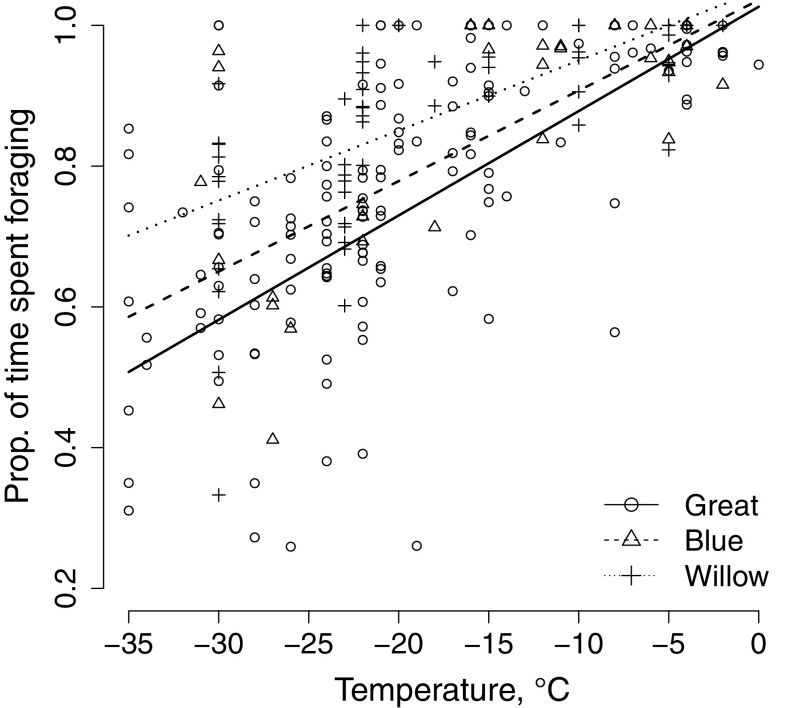
Relationship between the foraging rate (proportion of time spent in foraging) and temperature for blue tits, great tits, and willow tits

Overall foraging rates decreased with decreasing temperatures, and great tits and blue tits had lower foraging rates than willow tits (Fig. 1, Table 1). Foraging rate of great tits reduced significantly faster in response to decreasing temperatures when compared to the willow tit (Fig. 1, Table 1). The coefficients remained similar in the data spanning until − 30 °C (Online Resource 2).

**Table 1:** Best model estimates of parameters affecting foraging rates in blue tits, great tits, and willow tits in different temperatures (zT = centred temperature)

Parameter	Coefficient	SE	*df*	*Z*	*P*
Intercept	0.862	0.032	6.095	27.197	< 0.001
**zTemperature (zT)**	**0.180**	**0.037**	**229.657**	**4.898**	**< 0.001**
**Blue vs. willow**	**− 0.069**	**0.033**	**47.777**	**− 2.104**	**0.041**
**Great vs. willow**	**− 0.115**	**0.027**	**9.894**	**− 4.284**	**0.002**
Blue × zT vs. Willow × zT	0.053	0.056	215.426	0.933	0.352
**Great** × **zT vs. Willow** × **zT**	**0.088**	**0.044**	**230.728**	**2.025**	**0.044**

Random effects:	Variance	SD			
Site	0.0006	0.0250			
Year (2011)	0.0027	0.0524			
Year (2012)	0.0011	0.0328			
Residual	0.0174	0.1320			

Statistically significant (*p* ≤ 0.05) parameters are in bold. The analysis included 240 observations from two sites (Mid-Finland and Lapland)

### Warming and heat loss preventing behaviour

**Fig. 2:**
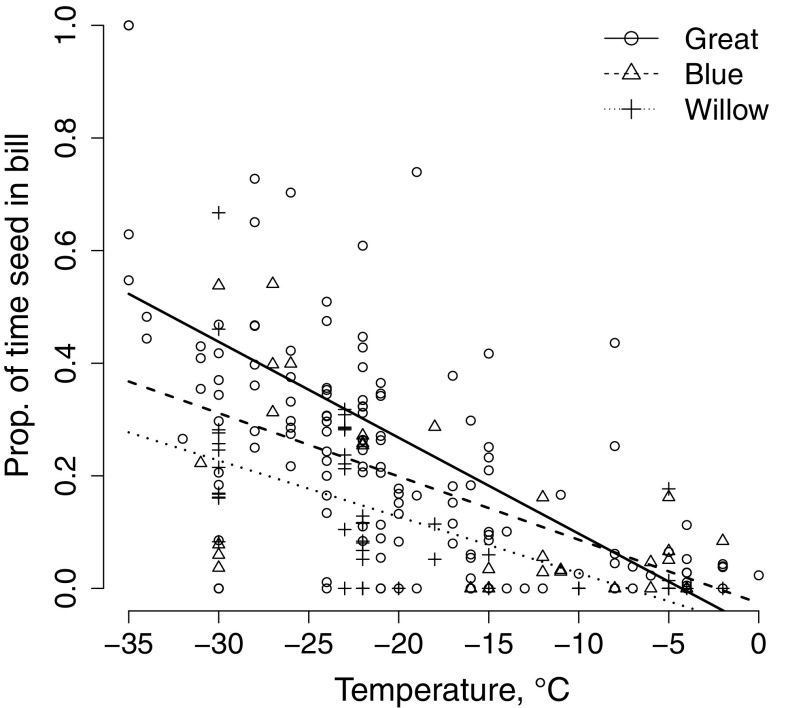
Relationship between warming behaviour (as indicated by proportion of time the seed was held in the bill) and temperature for blue tits, great tits, and willow tits

**Fig. 3:**
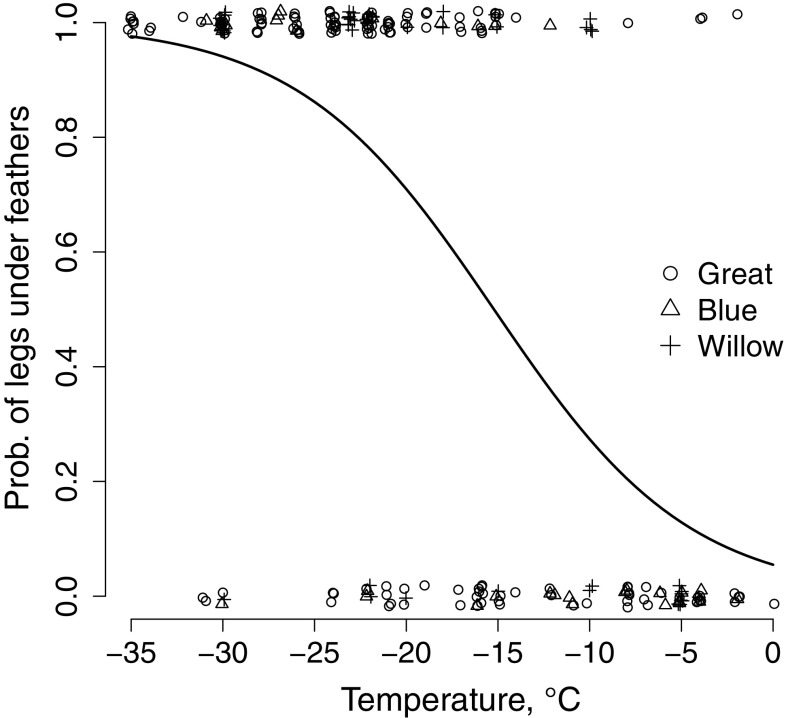
Relationship between heat loss preventing behaviour (legs under feathers or not, see details in methods) and temperature for blue tits, great tits, and willow tits

Warming behaviour increased in response to decreasing temperatures, and warming was more frequent in great tits and blue tits than in willow tits (Table 2; Fig. 2). Furthermore, the temperature response was stronger among great tits than in willow tits (Fig. 2; Table 2). This interaction was similar but did not remain significant in the data spanning until − 30 °C (Online Resource 2). Heat loss preventing behaviour (covering legs with feathers) increased in response to decreasing temperatures, but there were no between-species differences (Fig. 3; Table 3). The coefficient remained similar in the data spanning until − 30 °C (Online Resource 2).

**Table 2:** Best model estimates of parameters affecting warming behaviour (seed in the bill) in blue tits, great tits, and willow tits in different temperatures (zT = centred temperature)

Parameter	Coefficient	SE	*df*	*Z*	*P*
Intercept	0.115	0.033	5.375	3.504	0.015
**zTemperature (zT)**	**− 0.180**	**0.040**	**227.042**	** − 4.436**	**< 0.001**
**Blue vs. willow**	**0.070**	**0.036**	**45.164**	**1.972**	**0.055**
**Great vs. willow**	**0.132**	**0.029**	**9.531**	**4.560**	**0.001**
Blue × zT vs. Willow × zT	− 0.022	0.062	213.632	− 0.358	0.721
**Great** × **zT vs. Willow** × **zT**	**− 0.126**	**0.048**	**225.802**	**− 2.615**	**0.010**

Random effects:	Variance	SD			
Site	0.0007	0.0259			
Year (2011)	0.0034	0.0586			
Year (2012)	0.0007	0.0268			
Residual	0.0215	0.1466			

Statistically significant (*p* ≤ 0.05) parameters are in bold. The analysis included 237 observations from two sites (Mid-Finland and Lapland)

**Table 3:** Best model estimates of parameters affecting heat loss preventing behaviour (covering legs with feathers) in blue tits, great tits, and willow tits in different temperatures (T)

Parameter	Coefficient	SE	*Z*	*P*
Intercept	− 2.8473	0.0002	− 12905.000	< 0.001
**Temperature (** ***T*** **)**	**− 0.1871**	**0.0002**	**− 856.000**	**< 0.001**

Random effects:	Variance	SD		
Site	0.25446	0.5044		
Year (2011)	6.22E+06	2493.29		
Year (2012)	0.05848	0.2418		

Statistically significant (*p* ≤ 0.05) parameters are in bold. The analysis included 228 observations from two sites (mid-Finland and Lapland)

### Vigilance

**Fig. 4:**
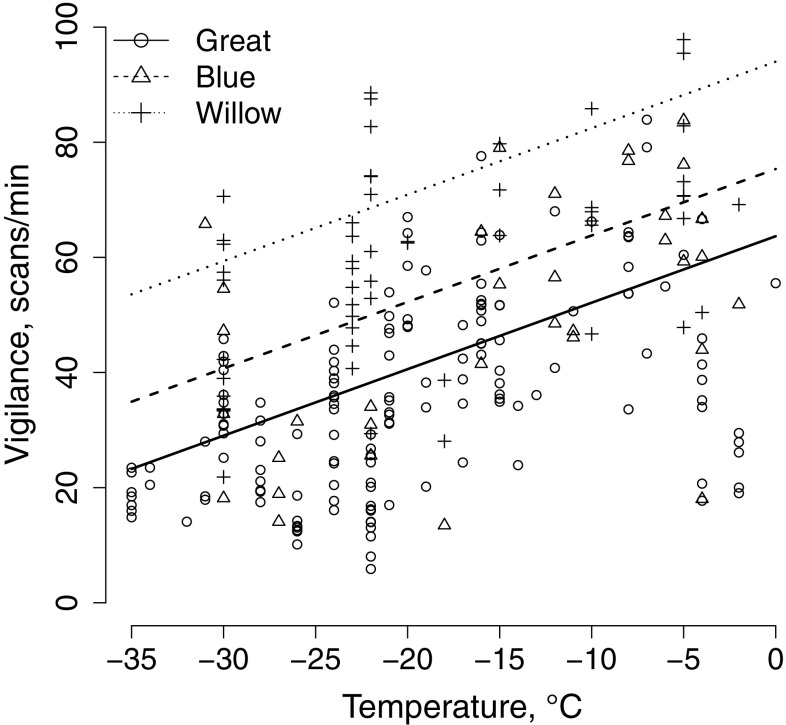
Relationships between vigilance (scans/min) and temperature for blue tits, great tits, and willow tits

Vigilance was reduced in response to decreasing temperature and consistently occurred less often in both blue tits and great tit compared to willow tits (Fig. 4; Table 4). The coefficient remained similar in the data spanning until − 30 °C (Online Resource 2).

**Table 4:** Best model estimates of parameters affecting vigilance in blue tits, great tits, and willow tits in different temperatures (*T*)

Parameter	Coefficient	SE	*df*	*Z*	*P*
Intercept	93.997	3.909	4.590	24.049	< 0.001
**Temperature (** ***T*** **)**	**1.155**	**0.093**	**213.500**	**12.448**	**< 0.001**
**Blue vs. willow**	**− 18.648**	**3.053**	**184.820**	**− 6.108**	**< 0.001**
**Great vs. willow**	**− 30.325**	**2.465**	**67.590**	**− 12.304**	**< 0.001**

Random effects	Variance	SD			
Site	260.808	16.150			
Year (2011)	0.004	0.065			
Year (2012)	52.058	7.215			
Residual	155.160	12.456			

Statistically significant (*p* ≤ 0.05) parameters are in bold. The analysis included 240 observations from two sites (mid-Finland and Lapland)

## Discussion

We found that foraging rates decreased, warming and heat loss preventing behaviour increased, and vigilance decreased in response to decreasing temperatures in all the species. As expected, the southern newcomers had lower foraging rates, more frequent warming behaviour, and were less vigilant compared to the better adapted boreal willow tits. It seems that even though wintering in the north is possible in terms of physiological aspects and available food, processing enough food to prevent death due starvation while escaping predation requires adaptations. These mechanisms acting in low temperatures may thus be important determinants of species winter ranges (Zuckerberg et al. 2011).

Willow tits had higher foraging rates than great tits and the decrease in the foraging rate in relation to lowering temperatures was stronger in the great tit than in the willow tit. In support of this, daily mass gain is the lowest during the coldest mid-winter months in great tits (Lehikoinen 1987), whereas willow tits do not show such variation (Broggi et al. 2003). While great tits can somewhat benefit from the physiological and thermal benefits of their larger size (Lehikoinen 1986), reduced foraging rates can be a strain especially for the great tit which weighs nearly twice that of a willow tit, because energy consumption increases with size of the animal (McNab 1971; Calder and King 1974). Foraging was disturbed by warming behaviour when temperatures declined in all the species, but this change was stronger among great tits than willow tits. This result provides an explanation for the foraging rate difference in low temperatures, and suggests different seasonal adaptations to heat production in northern species (Carlson et al. 1993; Broggi et al. 2007; Petit et al. 2013) or species-specific cold tolerance and insulation properties of their plumage (Saarela et al. 1995). Indeed, great tits are unable to produce optimal feathers in northern conditions due to time and/or nutrient constraints, and this may lead to a plumage with poorer insulation properties (Broggi et al. 2011). Species differences in response to temperature may be evident only in the coldest temperatures as the interaction became less evident with the reduced data. This may also reflect the lack of data (and thus lower statistical power) for the complicated models that include the random effects of site with random slopes for years.

While blue tits had lower foraging rates and spent more time warming than willow tits, their responses to temperature were similar. It is possible that these behaviours are somewhat linked to morphology. Willow tits and blue tits have shorter feet and bills than great tits (Partridge 1976; Norberg 1979), which may reduce heat loss (Cardilini et al. 2016). Shorter legs also enable them to keep their centre of mass closer to the branch, which makes keeping the posture easier (Norberg 1979) and allows foraging even when their feet are almost covered by their plumage (own observations).

Heat loss preventing behaviour increased in response to cold temperatures similarly in all species. Birds may avoid heat loss preventing postures such as keeping legs within the plumage that can reduce reaction times to predation (Carr and Lima 2011), and use them mostly under extreme conditions when maintaining body or peripheral (leg) temperature becomes more important than escaping predation. In line with this, we found a threshold for feet warming (ca. − 20 °C) below which this behaviour increased (Fig. 3). Birds have to cover their legs as heat loss becomes too high either for maintaining deep body temperature or for preventing freeze damage via the counter-current heat exchange between the arteries and veins. Energetically, it is cheaper to prevent cold damage by hiding the feet in feathers than by increasing the flow of arterial warm blood as this would further increase heat loss.

Vigilance reduced in all species when temperatures decreased. This result was similar to the previous studies (Caraco 1974; Hogstad 1988a, 2015; Pravosudov and Grubb 1995); nevertheless, great tits and blue tits scanned less than willow tits. This may be due to the better cold tolerance and higher foraging rate of willow tits. Information on food hoards may also reduce stress of accessing food, and thereby leave more time for vigilance.

Our results suggest that especially great tits have not fully adapted to the northern conditions, which is consistent with the previous studies on breeding great tits (Veistola et al. 1995; Rytkönen and Orell 2001; Krama et al. 2013). Birds respond to the cold by increasing metabolism, i.e., basal and summit metabolic rates (e.g., Petit et al. 2013). Resting great tits, for example, increase their metabolic rate to 3 × BMR at − 20 °C (Broggi et al. 2004, 2007), which requires the consumption of more food. However, during cold spells reaching − 35 °C, their foraging rate was reduced to nearly half. Low foraging rate may, therefore, be detrimental, especially when food is scarce and the day length is short. Great tits also move less during mid-winter in cold temperatures (Pakanen et al. 2018), which can make it even more difficult to find enough food. As a result, cold weather may result in starvation, stress, and eventually death (Krams et al. 2010, 2013). This is exacerbated by the poorer and reduced ability to keep vigilant in cold weather, making them vulnerable to predation (Morosinotto et al. 2017). It is, therefore, possible that cold temperatures limit success of great tits wintering in northern conditions by increasing mortality (Järvinen 1983). This conjecture is supported by an effect of winter temperature on annual survival (Perdeck et al. 2000) and the lower survival of breeding great tits in the north (0.38; Karvonen et al. 2012) compared to sympatric willow tits (0.59; Lampila et al. 2006) but also compared to great tits breeding in Central and Southern Europe (ca. 0.5; Payevsky 2006).

Here, we show that the southern newcomers wintering at high latitudes are not adapted to the cold temperatures in terms of foraging behaviour. Even when fed with supplemental food, their foraging rates and vigilance decreased in cold ambient temperatures and were lower than in a boreal species which is better adapted to the northern conditions. Climate change will undoubtedly help southern species winter at higher latitudes via processes such as earlier arrival of spring, increased proportion of bare ground, and less snow cover. Our results suggest that, if climate change increases winter temperatures and especially reduces the length and severity of cold spells in the northern latitudes, southern species will benefit from increased foraging rates and vigilance. These should result in higher survival, stronger population growth, and consequent range expansion towards the north.

## Electronic supplementary material

Below is the link to the electronic supplementary material.

Supplementary material 1 (PDF 348 kb)

Supplementary material 2 (PDF 266 kb)
