# Supplementary material for: Northward expanding resident species benefit from warming winters through increased foraging rates and predator vigilance
Source: Oecologia. 2018 Oct 24;188(4):991–9. doi: 10.1007/s00442-018-4271-7 (PMC6244859; doi:10.1007/s00442-018-4271-7)
Supplement: Supplementary file 1 — Supplementary material 1 (PDF 348 kb) [file 442_2018_4271_MOESM1_ESM.pdf]

Electronic Supplemental Material 1

Pakanen V-M, Ahonen E, Hohtola E & Rytkönen S (2018) Northward expanding resident species benefit from warming winters through increased foraging rates and predator vigilance. Oecologia

Table S1. Description of recording lengths and temperatures during recording for each species.

	Temperature (°C)			Recording lenghts (seconds)			
Species	mean	SD	Range	mean	SD	Range	n
Blue tit	-15.4	9.7	(-31) - (-2)	37.8	36.2	10.9 - 182.9	34
Great tit	-19.7	8.8	(-35) - (0)	39.3	28.2	10.1 - 146.9	152
Willow tit	-18.9	8.9	(-30) - (-2)	34.9	26.6	13.2 - 192.5	55
