# Supplementary material for: Northward expanding resident species benefit from warming winters through increased foraging rates and predator vigilance
Source: Oecologia. 2018 Oct 24;188(4):991–9. doi: 10.1007/s00442-018-4271-7 (PMC6244859; doi:10.1007/s00442-018-4271-7)
Supplement: Supplementary file 2 — Supplementary material 2 (PDF 266 kb) [file 442_2018_4271_MOESM2_ESM.pdf]

Electronic Supplemental Material 2

Pakanen V-M, Ahonen E, Hohtola E & Rytkönen S (2018) Northward expanding resident species benefit from warming winters through increased foraging rates and predator vigilance. Oecologia

Tables S2 - S5 tabulate the coefficients and their SE of statistical models explaining variation in foraging rates, warming behaviour, heat loss preventing behaviour and vigilance that were applied to all data (see also Tables 1 - 4) and full model results from models that were applied to data spanning from -30 to 0 °C.

Table S2. Comparison of model estimates of parameters affecting foraging rates in blue tits, great tits and willow tits derived from all data and reduced data spanning from -30 to 0 °C.

	All data (n = 240)		Reduced data (n = 226)				
Parameter	Coeff	SE	Coeff	SE	df	z	p
Intercept	0.862	0.032	0.869	0.028	14.063	30.953	<0.001
**zTemperature (zT)**	**0.180**	**0.037**	**0.170**	**0.034**	**214.904**	**4.970**	**<0.001**
**Blue Vs. Willow**	**-0.069**	**0.033**	**-0.070**	**0.033**	**152.940**	**-2.126**	**0.035**
**Great Vs. Willow**	**-0.115**	**0.027**	**-0.113**	**0.029**	**49.532**	**-3.936**	**<0.001**
Blue x zT vs. Willow x zT	0.053	0.056	0.058	0.054	216.408	1.089	0.277
**Great x zT vs. Willow x zT**	**0.088**	**0.044**	*0.083*	*0.042*	*217.449*	*1.963*	*0.051*
Random effects:	Variance	SD	Variance	SD			
Site	0.0006	0.0250	0.0000	0.0000			
Year(2011)	0.0027	0.0524	0.0010	0.0312			
Year(2012)	0.0011	0.0328	0.0033	0.0578			
Residual	0.0174	0.1320	0.0170	0.1304			

Table S3. Comparison of model estimates of parameters affecting warming behaviour (seed in the bill) in blue tits, great tits and willow tits derived from all data and reduced data spanning from -30 to 0 °C.

	All data (n = 237)		Reduced data (n = 225)				
Parameter	Coeff	SE	Coeff	SE	df	z	p
Intercept	0.115	0.033	0.110	0.029	9.597	3.836	0.004
**zTemperature (zT)**	**-0.180**	**0.040**	**-0.172**	**0.035**	**213.371**	**-4.913**	**<0.001**
**Blue Vs. Willow**	**0.070**	**0.036**	**0.072**	**0.034**	**140.937**	**2.131**	**0.035**
**Great Vs. Willow**	**0.132**	**0.029**	**0.117**	**0.029**	**40.730**	**3.994**	**<0.001**
Blue x zT vs. Willow x zT	-0.022	0.062	-0.025	0.055	215.284	-0.449	0.654
**Great x zT vs. Willow x zT**	**-0.126**	**0.048**	-0.061	0.043	215.837	-1.419	0.157
Random effects:	Variance	SD	Variance	SD			
Site	0.0007	0.0259	0.0000	0.0000			
Year(2011)	0.0034	0.0586	0.0007	0.0262			
Year(2012)	0.0007	0.0268	0.0030	0.0545			
Residual	0.0215	0.1466	0.0178	0.1332			

Table S4. Comparison of model estimates of parameters affecting affecting heat loss preventing behaviour (covering legs with feathers) in blue tits, great tits and willow tits derived from all data and reduced data spanning from -30 to 0 °C.

	All data (n = 228)		Reduced data (n = 214)			
Parameter	Coeff	SE	Coeff	SE	z	p
**Intercept**	**-2.8473**	**0.0002**	**-2.947**	**0.000**	**-27211.000**	**<0.001**
**Temperature (T)**	**-0.1871**	**0.0002**	**-0.198**	**0.000**	**-1832.000**	**<0.001**
Random effects:	Variance	SD	Variance	SD		
Site	0.25446	0.5044	0.16235	0.4029		
Year(2011)	6.22E+06	2493.29	2.11E+06	4596.37		
Year(2012)	0.05848	0.2418	0.09865	0.3141		

Table S5. Comparison of model estimates of parameters affecting affecting vigilance in blue tits, great tits and willow tits derived from all data and reduced data spanning from -30 to 0 °C.

	All data (n = 240)		Reduced data (n = 226)				
Parameter	Coefficient	SE	Coefficient	SE	df	z	p
Intercept	93.997	3.909	91.919	3.823	5.346	24.043	<0.001
**Temperature (T)**	**1.155**	**0.093**	**1.091**	**0.101**	**205.640**	**10.816**	**<0.001**
**Blue Vs. Willow**	**-18.648**	**3.053**	**-19.165**	**3.113**	**177.289**	**-6.157**	**<0.001**
**Great Vs. Willow**	**-30.325**	**2.465**	**-29.522**	**2.527**	**61.000**	**-11.683**	**<0.001**
Random effects:	Variance	SD	Variance	SD			
Site	260.808	16.150	260.273	16.133			
Year(2011)	0.004	0.065	1.711	1.308			
Year(2012)	52.058	7.215	59.123	7.689			
Residual	155.160	12.456	158.072	12.573			
